# Physical interactions in non-ideal fluids promote Turing patterns

**DOI:** 10.1098/rsif.2023.0244

**Published:** 2023-07-12

**Authors:** Lucas Menou, Chengjie Luo, David Zwicker

**Affiliations:** Max Planck Institute for Dynamics and Self-Organization, Am Faßberg 17, Göttingen 37077, Germany

**Keywords:** reaction–diffusion systems, Turing patterns, phase separation, non-ideal systems

## Abstract

Turing’s mechanism is often invoked to explain periodic patterns in nature, although direct experimental support is scarce. Turing patterns form in reaction–diffusion systems when the activating species diffuse much slower than the inhibiting species, and the involved reactions are highly nonlinear. Such reactions can originate from cooperativity, whose physical interactions should also affect diffusion. We here take direct interactions into account and show that they strongly affect Turing patterns. We find that weak repulsion between the activator and inhibitor can substantially lower the required differential diffusivity and reaction nonlinearity. By contrast, strong interactions can induce phase separation, but the resulting length scale is still typically governed by the fundamental reaction–diffusion length scale. Taken together, our theory connects traditional Turing patterns with chemically active phase separation, thus describing a wider range of systems. Moreover, we demonstrate that even weak interactions affect patterns substantially, so they should be incorporated when modelling realistic systems.

## 1. Introduction

Natural periodic patterns, ranging from nano-crystals [1], tissues [2], populations dynamics [3], to geophysical phenomena [4], are often explained by the seminal Turing mechanism [5–8]. Turing patterns generally describe the spatial distribution of an activator and an inhibitor that diffuse in space. Patterns then form when the localized activator triggers production while the inhibitor suppresses production globally, often summarized as *local activation, global inhibition* [6,7]. However, it is not clear whether Turing’s mechanism can actually explain natural patterns [9,10], since inhibitors need to diffuse much faster than activators and the involved reactions need to be highly nonlinear [11–13]. Such nonlinear reactions are often motivated by cooperative reactions, where multiple reactants lead to nonlinearities [12]. Cooperativity typically originates from physical interactions, which should also affect the diffusive motion of the species, but this is typically not taken into account.

Physical interactions are crucial for organizing biomolecules in cells [14], cells in tissues [15] and even organisms in groups [16]. In particular, multivalent interactions can induce phase separation, where a dense droplet phase segregates spontaneously from a dilute surrounding phase. This is possible since the enthalpic gain from the interactions overcompensates the entropic loss of concentrating constituents [17,18]. In simple passive systems, surface tension implies that large droplets grow to system size at the expense of small droplets [19]. However, chemical reaction can suppress this Ostwald ripening [20] and thus control the size and arrangement of droplets [21–23]. The predicted hexagonal arrangements [24] are very reminiscent of Turing patterns, although patterns are driven by phase separation in these systems. Taken together, although droplets regulated by chemical reactions share some properties with Turing patterns, it is unclear how the two models are related.

In this paper, we study a minimal system that is capable of forming droplets as well as Turing patterns. Effectively, we add physical interactions between activator and inhibitor to a standard reaction–diffusion system. This approach allows us to quantify how interactions affect pattern formation and it unveils a range of systems that interpolate between Turing’s mechanism and patterns formed in active phase separating systems. In particular, we show how weak repulsive interactions stabilize patterns by inducing cross-diffusion, while strong interactions lead to phase separation, where coarsening is arrested by chemical reactions.

## 2. Results

### 2.1. Interactions affect pattern formation

We start by considering a minimal pattern forming system comprising two species: an activator *A* and an inhibitor *I*. The basic Turing model describes the dynamics of the respective fractions $\phi_{A}(r,t)$ and $\phi_{I}(r,t)$ as a function of the spatial position r and time *t*,

2.1 $$\partial_{t}\phi_{i}=\sum_{\;j=A,I}D_{ij}\nabla^{2}\phi_{j}+k\left[\frac{2\phi_{0}}{1+{\left(\phi_{I}/\phi_{A}\right)}^{h}}-\phi_{i}\right]$$

for *i* = *A*, *I*. Here, the first term on the right-hand side describes ideal diffusion with a diffusivity matrix $D_{ij}$ and the second term captures chemical reactions based on the Hill–Langmuir equation [25]. For *h* ≥ 1, these reactions promote the production of *A* and *I* by activator *A* and suppress it by the inhibitor *I*, while both species exhibit linear degradation with rate *k*. This choice of chemical reactions allows us to independently control the typical fraction *ϕ*_0_ of components *A* and *I*, the reaction rate *k*, and the reaction nonlinearity *h*. We show in the electronic supplementary material that equation (2.1) exhibits a Turing instability for sufficiently large *h* when the inhibitor *I* diffuses faster than the activator *A*, so *I* spreads out while *A* stays localized.

**Figure 1.**
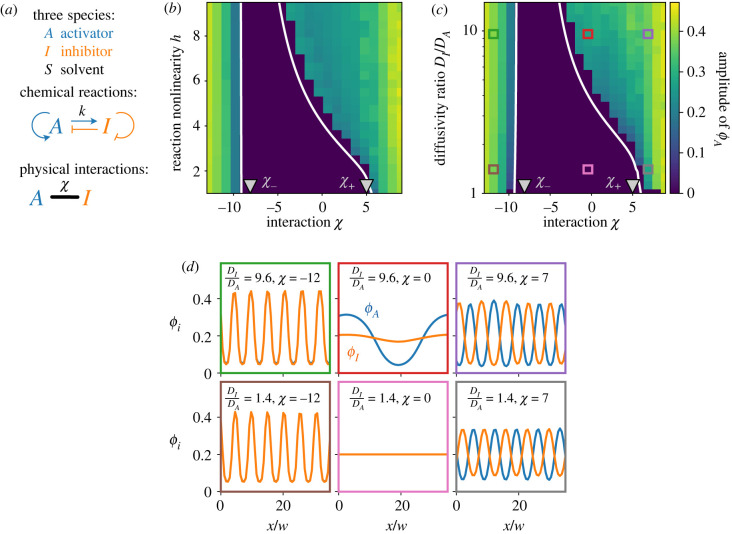
Interactions affect pattern formation. (*a*) Schematic of chemical and physical interactions of activator *A*, inhibitor *I* and the inert solvent. (*b*) Stationary state amplitudes of fraction *ϕ*_*A*_ as a function of the interaction strength *χ* and the reaction nonlinearity *h* for diffusivity ratio *D*_*I*_/*D*_*A*_ = 5. (*c*) Amplitude as a function of *χ* and *D*_*I*_/*D*_*A*_ for *h* = 5. (*d*) Stationary patterns of *ϕ*_*A*_ (blue) and *ϕ*_*I*_ (orange) for the indicated parameters. (*b*,*c*) The homogeneous state is stable between the white lines, obtained from a linear stability analysis of equation (2.4), and the grey triangles mark critical interaction values *χ*_−_ and *χ*_+_; see electronic supplementary material. (*b*–*d*) Model parameters are *k* = 0.1 *D*_*A*_/*w*^2^ and *ϕ*_0_ = 0.2. Simulations ran for *t* = 10^5^ *w*^2^/*D*_*A*_ on a one-dimensional grid of length 200 *w* with periodic boundary conditions.

To include physical interactions between activator *A* and inhibitor *I*, we first consider the thermodynamics of an incompressible, isothermal fluid comprising the species *A* and *I* as well as an inert solvent *S*; see figure 1*a*. This system is still fully described by the volume fractions *ϕ*_*A*_ and *ϕ*_*I*_, since the solvent occupies the remaining fraction *ϕ*_*S*_ = 1 − *ϕ*_*A*_ − *ϕ*_*I*_. The interactions of *A* and *I* in such a fluid can then be described by the Flory–Huggins free energy [26–28]

2.2 $$\begin{array}{cc} F[\phi_{A},\;\phi_{I}]\; & =\frac{k_{B}T}{\nu }\int [\phi_{A}\ln\phi_{A}+\phi_{I}\ln\phi_{I}+\phi_{S}\ln\phi_{S}+\chi \phi_{A}\phi_{I}.\;\\ \; & \;+\frac{w^{2}}{2}\;\left(|\nabla \phi_{A}{|}^{2}+|\nabla \phi_{I}{|}^{2}\right)]dr,\; \end{array}$$

where the integral is over the volume of the system, *k*_*B*_ *T* is the relevant energy scale and *ν* denotes a molecular volume, which we assume to be the same for all species. The first three terms in the square bracket capture the translational entropies of all species, the fourth term describes the physical interaction between *A* and *I*, and the last term limits the width of interfaces between coexisting phases to roughly *w* in strongly interacting systems [28]. The interactions between *A* and *I* are quantified by the Flory parameter *χ*: positive *χ* denotes effective repulsion, which can originate from heterotypic repulsion or homotypic attraction, while negative *χ* leads to attraction of *A* and *I*. Equilibrium states, which minimize the free energy given by equation (2.2), can be inhomogeneous when interactions are sufficiently strong [29]: for strong attraction (large negative *χ*), a phase enriched in *A* and *I* will segregate from one enriched in the solvent, whereas strong repulsion (large positive *χ*) will lead to segregation of *A* from *I* with an equal amount of solvent in both phases. However, it is unclear how this equilibrium behaviour is modified by the active reactions described by equation (2.1) and how Turing patterns are affected by weak interactions.

To model reaction–diffusion dynamics with interactions described by the Flory parameter *χ*, we replace the ideal diffusion term in equation (2.1) by a more general form which describes diffusion in non-ideal fluids. Linear non-equilibrium thermodynamics implies that diffusive fluxes are then proportional to gradients of the chemical potentials associated with the free energy given by equation (2.2), and the proportionality constants (known as Onsager coefficients or mobilities) determine the kinetic rate [30,31]. Defining non-dimensional exchange chemical potentials *μ*_*i*_ = *ν*(*k*_*B*_ *T*)^−1^*δF*/*δϕ*_*i*_,

2.3a $$\mu_{A}=\ln(\phi_{A})-\ln(\phi_{S})+\chi \phi_{I}-w^{2}\nabla^{2}\phi_{A}$$

and

2.3b $$\mu_{I}=\ln(\phi_{I})-\ln(\phi_{S})+\chi \phi_{A}-w^{2}\nabla^{2}\phi_{I},$$

we thus find

2.4 $$\partial_{t}\phi_{i}=\nabla \cdot (D_{i}\phi_{i}\nabla \mu_{i})+k\left[\frac{2\phi_{0}}{1+{\left(\phi_{I}/\phi_{A}\right)}^{h}}-\phi_{i}\right],$$

where *D*_*i*_ are the diffusivities of the species *i* = *A*, *I*, which are related to the mobilities *D*_*i*_*ϕ*_*i*_ in this multi-component system [32]. We show in the electronic supplementary material that equation (2.4) reduces to equation (2.1), and thus describes ideal diffusion, if physical interactions are absent (*χ* = 0) and the wavelength of patterns is large compared with *w*. Consequently, (2.4) describes a reaction–diffusion system encompassing non-ideal diffusion and containing normal Turing patterns as a limiting case.

To see how interactions affect patterns, we performed numerical simulations of equations (2.3) and (2.4) in a one-dimensional system with periodic boundary conditions; see Methods. Figure 1 demonstrates that without interactions (*χ* = 0), patterns with finite amplitudes only emerge if the reactions are sufficiently nonlinear (large *h*) and the inhibitor diffuses sufficiently fast (*D*_*I*_ ≫ *D*_*A*_), as expected for Turing patterns [5]. This trend persists for weak interactions, although the corresponding threshold values of *h* and *D*_*I*_/*D*_*A*_ change. Apparently, repulsion between *A* and *I* promotes pattern formation (*χ* > 0), while attraction suppresses it (*χ* < 0). However, very strong attraction can again lead to large amplitudes (χ≲−9), independent of *h* and *D*_*I*_/*D*_*A*_.

The corresponding volume fraction profiles shown in figure 1*d* corroborate these observations: without interactions (middle column), the system stays either homogeneous (pink parameter set) or forms normal Turing patterns (red parameter set) with a localized activator *A* and a fairly homogeneous inhibitor *I*. By contrast, strong attraction (left column) leads to co-localization of *A* and *I*, reminiscent of phase separation, albeit with a well-defined pattern length scale. Similarly, *A* segregates from *I* for strong repulsion (right column). Taken together, we thus showed that there is an interesting interplay between stereotypical Turing patterns and interactions promoting phase separation.

### 2.2. Weak interactions imply cross-diffusion

To understand how interactions affect pattern formation, we first analyse weak interactions (χ≲5) by treating them as a perturbation to normal Turing patterns. Assuming the wavelength of patterns is large compared with *w*, the generalized diffusion in equation (2.4) can be approximated by ideal diffusion to first order in *χ*; see electronic supplementary material. Consequently, the dynamics are described by equation (2.1) with the diffusivity matrix

2.5 $$D_{ij}\approx \left(\begin{array}{cc} D_{A}(1+\psi ) & D_{A}(\psi +\chi \phi_{0})\\ D_{I}(\psi +\chi \phi_{0}) & D_{I}(1+\psi ) \end{array}\right),$$

where *ψ* = *ϕ*_0_/(1 − 2*ϕ*_0_). This analysis demonstrates that without interactions (*χ* = 0) in dilute system (*ϕ*_0_ ≪ 1, avoiding crowding effects), diffusion is dominated by the diagonal entries, resulting in stereotypical Turing patterns. In this case, we show analytically in the electronic supplementary material that patterns can only form when the diffusivity ratio *D*_*I*_/*D*_*A*_ and the reaction nonlinearity *h* are sufficiently large, consistent with the literature [12,13].

**Figure 2.**
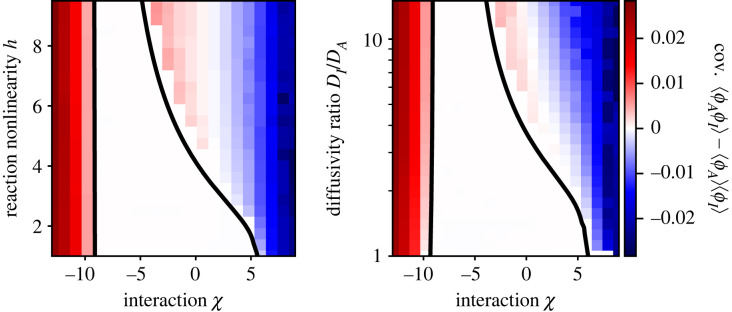
Interactions affect correlation between activator and inhibitor. Covariance 〈*ϕ*_*A*_*ϕ*_*I*_〉 − 〈*ϕ*_*A*_〉〈*ϕ*_*I*_〉 as a function of interaction *χ* and reaction nonlinearity *h* (left, *D*_*I*_/*D*_*A*_ = 5) or diffusivity ratio *D*_*I*_/*D*_*A*_ (right, *h* = 5). The homogeneous state is stable between the black lines, obtained from a linear stability analysis of equation (2.4); see electronic supplementary material. Model parameters are *k* = 0.1 *D*_*A*_/*w*^2^ and *ϕ*_0_ = 0.2. Simulations ran for *t* = 10^5^ *w*^2^/*D*_*A*_ on a periodic one-dimensional grid of length 200 *w*.

**Figure 3.**
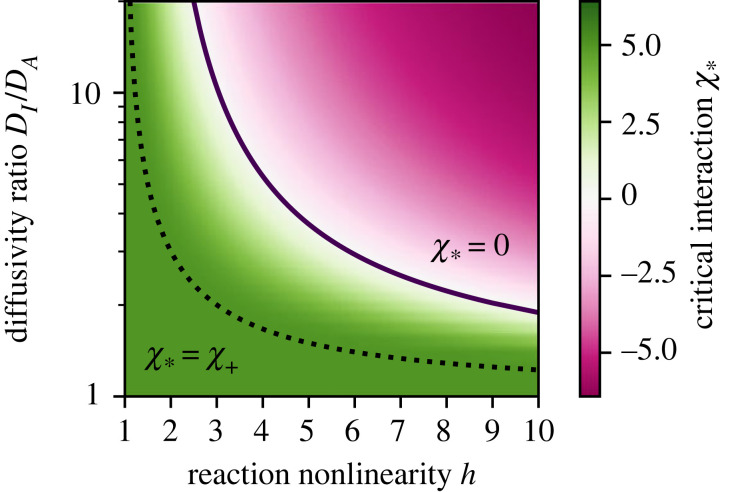
Repulsive interactions improve trade-off between differential diffusivity and reaction nonlinearity. Minimal interaction $\chi_{*}$ to support patterns (equation S11 in the electronic supplementary material) as a function of diffusivity ratio *D*_*I*_/*D*_*A*_ and reaction nonlinearity *h* for *ϕ*_0_ = 0.2. Turing patterns without interactions ($\chi_{*}=0$) form above the solid line. Conversely, phase separation is required to form patterns below the dotted line ($\chi_{*}>\chi_{+}=5$).

If *A* and *I* interact (*χ* ≠ 0), equation (2.5) reveals that interactions directly affect cross-diffusion of *A* and *I*. For example, repulsive interactions (*χ* > 0) imply fluxes of *A* opposite to the gradient of *I*, thus favouring the segregation of the two species and enhancing patterns [33]. To quantify this behaviour, we analyse the covariance, cov(*ϕ*_*A*_, *ϕ*_*I*_) = 〈*ϕ*_*A*_*ϕ*_*I*_〉 − 〈*ϕ*_*A*_〉〈*ϕ*_*I*_〉, where the brackets denote spatial averages in the stationary state. Figure 2 shows that the covariance generally decreases with more repulsive interactions (larger *χ*, consistent with enhanced cross-diffusion), increasing reaction nonlinearity *h*, and diffusivity ratio *D*_*I*_/*D*_*A*_. The more detailed stability analysis presented in the electronic supplementary material demonstrates that repulsive interactions always promote pattern formation and lower the required reaction nonlinearity *h* and diffusivity ratio *D*_*I*_/*D*_*A*_; see figure 3. By contrast, attractive interactions (*χ* < 0) generally stabilize the homogeneous system. However, this behaviour only holds for moderate interactions *χ* since strong attraction (χ≲−9) also leads to large amplitudes; see figure 1.

### 2.3. Strong interactions invoke phase separation

Turing’s mechanism cannot explain patterns that form when the activator *A* and inhibitor *I* attract each other strongly (*χ* < −9). Based on the strong correlations between *A* and *I* seen in figure 2, we hypothesize that strong attraction leads to associative phase separation of *A* and *I* from the solvent, while the reactions play a minor role. Indeed, we show in the electronic supplementary material that phase separation is possible in the absence of reactions when *χ* < *χ*_−_, where *χ*_−_ = 8 arctanh(1 − 4*ϕ*_0_)/(4*ϕ*_0_ − 1) marks the binodal point for a given average fraction *ϕ*_0_ of *A* and *I*. Figure 1 shows that the value *χ*_−_ is very close to the onset of patterns, and that the resulting profiles are perfectly co-localized. Taken together, strong attraction between *A* and *I* leads to co-segregation of the two components from the solvent while the reactions barely affect the amplitude.

Strong repulsion between *A* and *I* should also lead to phase separation. In fact, for *χ* > *χ*_+_ with *χ*_+_ = 1/*ϕ*_0_, we predict that *A* can segregate from *I* spontaneously, even without reactions present; see electronic supplementary material. Figures 1 and 2 suggest that increasing the repulsion beyond this point indeed results in strong anti-correlation between *A* and *I* and a vanishing threshold for *h* and *D*_*I*_/*D*_*A*_. These numerical data indicate a continuous transition from patterns formed by reactions and diffusion (Turing patterns for weak interactions) to those formed by phase separation (strong interactions, *χ* < *χ*_−_ or *χ* > *χ*_+_). Taken together, we showed that patterns can form by reactions and by phase separation with an intricate interplay between them.

### 2.4. Reaction rate controls pattern length scale

**Figure 4.**
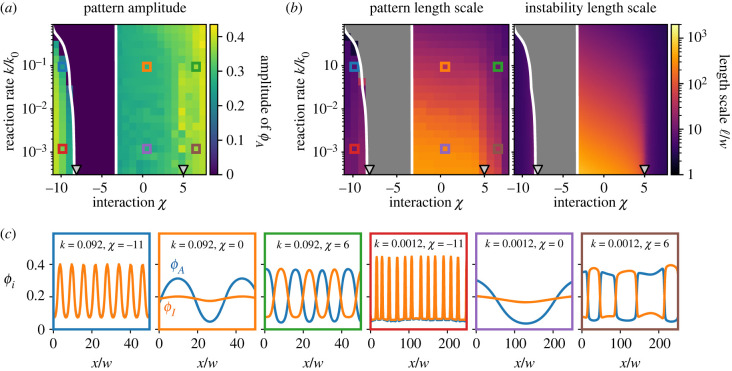
Reaction rate *k* determines pattern length scale. (*a*) Amplitude of activator *ϕ*_*A*_ as a function of interaction *χ* and reaction rate *k*. (*b*) Pattern length scale ℓ determined from the maximum of the structure factor of *ϕ*_*A*_ from numerical simulations (left) and from the fast growing mode in a linear stability analysis (right) as a function of *χ* and *k*. (*c*) Stationary patterns of *ϕ*_*A*_ (blue) and *ϕ*_*I*_ (orange) for various parameters indicated in (*a*,*b*). (*b*,*c*) The homogeneous state is stable between the white lines, obtained from a linear stability analysis of equation (2.4), and the grey triangles mark critical interaction values *χ*_−_ and *χ*_+_; see electronic supplementary material. (*a*–*c*) Model parameters are *h* = 5, *D*_*I*_/*D*_*A*_ = 10, *ϕ*_0_ = 0.2 and *k*_0_ = *D*_*A*_/*w*^2^. Simulations ran for $t=10^{7}k_{0}^{-1}$ on a periodic one-dimensional grid of length 2000 *w*.

We next ask what determines the length scale ℓ of the patterns. We show in the electronic supplementary material that ℓ is hardly affected by variations of the reaction nonlinearity *h* and diffusivity ratio *D*_*I*_/*D*_*A*_. By contrast, the interaction strength *χ* has a stronger influence: More repulsive interactions lead to patterns with shorter wavelengths, presumably because larger *χ* promote pattern formation. However, the strongest influence on the pattern length scale ℓ is the reaction rate *k*: numerical simulations and the linear stability analysis presented in figure 4 indicated that *k* allows adjusting ℓ over several orders of magnitude with barely any changes in the pattern amplitude.

To understand how the reaction rate *k* affects the pattern length scale ℓ, we first focus on weak interactions. In this case, interactions mainly cause cross-diffusion (see equation (2.5) and electronic supplementary material), implying that the reaction–diffusion lengths $\sqrt{D_{A}/k}$ and $\sqrt{D_{I}/k}$ are the only length scales in the equations. Consequently, length scales in the stationary state and in the initial instability must scale with *k*^−1/2^ for weak interactions, consistent with figure 4*b*. For strong interactions (*χ* < *χ*_−_ or *χ* > *χ*_+_), the system exhibits phase separation, implying that the initial instability is dominated by short patterns of length *w* while the stationary state patterns may exhibit much longer length scales due to coarsening [34]. Figure 4*b* shows that the linear stability analysis indeed predicts ℓ ∼ *w* in the region where we predict phase separation. For associative phase separation at strong attraction (*χ* < *χ*_−_), these patterns remain stable, and coarsening is suppressed; the variation in ℓ reflects the influence of *χ* on the interfacial width; see electronic supplementary material. In the contrasting case of strong repulsion (*χ* > *χ*_+_), patterns coarsen to the reaction–diffusion length and thus scale with *k*^−1/2^; see figure 4*b*. This behaviour is similar to the coarsening observed in active droplets, where the final length scale is also governed by the reaction–diffusion length [20]. Note that the numerical data presented in figure 4*b* might not represent the full stationary state since domain sizes only grow logarithmically with time in these one-dimensional systems [34]. However, our analysis demonstrates that the length scale ℓ of the final pattern is generally governed by reaction–diffusion lengths, except when strong attraction between *A* and *I* leads to associative phase separation.

### 2.5. Results generalize to higher dimensions

**Figure 5.**
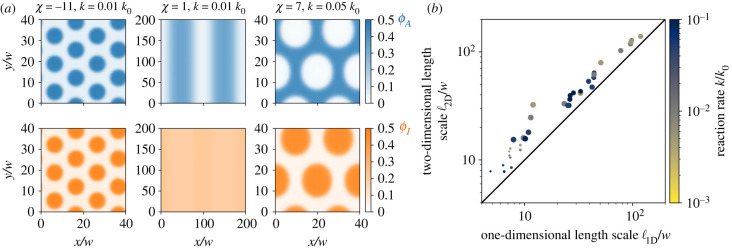
Interactions also control patterns in higher dimensions. (*a*) Two-dimensional stationary patterns of *ϕ*_*A*_ (upper panels) and *ϕ*_*I*_ (lower panels) for strong attraction (*χ* = −11), weak interaction (*χ* = 1) and strong repulsion (*χ* = 7) from left to right. (*b*) Correlation of length scales ℓ determined from numerical simulations in one and two dimensions for various reaction rates *k* (colour scale) and interactions *χ* (marker size). (*a*,*b*) Additional model parameters are *h* = 5, *D*_*I*_/*D*_*A*_ = 10, *ϕ*_0_ = 0.2 and *k*_0_ = *D*_*A*_/*w*^2^.

So far, we have focused on pattern formation in one dimension for simplicity, but many natural patterns form in planar geometries. To see whether our results hold for this relevant case, we next perform a few selected simulations in two dimensions; see figure 5. Analogously to one dimension, we find Turing patterns for weak interactions (middle column of figure 5*a*) and strong interactions induce phase separation. In particular, strong attraction between *A* and *I* leads to co-localization (left column) whereas strong repulsion induces anti-correlated patterns (right column). Interestingly, in both cases of phase separation droplets form instead of stripe patterns, even though both phases occupy roughly half of the space. In such a case, normal phase separating systems exhibit stripe patterns [35], but the reaction–diffusion dynamics in our system apparently alter the picture. In any case, figure 5*b* shows that the pattern length scales we measured in one dimension are very close to the ones measured in two-dimensional simulations for the same parameters, suggesting that the results from the simple one-dimensional system translate to the more complex two-dimensional system and also hold in higher dimensions.

## 3. Discussion

We propose an extension to Turing patterns that takes into account physical interactions that occur naturally. Weak repulsion between activator and inhibitor enhances patterns by inducing cross-diffusion, thus amplifying local activation and global inhibition. By contrast, strong interactions lead to phase separation, which can either be associative (*A* and *I* co-localize) or segregative (*A* separates from *I*). Both cases exhibit patterns for a much larger range of diffusivities and reaction nonlinearities than normal Turing patterns, and the resulting length scales differ: in the segregative case of strong repulsion, patterns are governed by the reaction–diffusion length scale and thus grow larger for weaker reactions. By contrast, patterns in the associative case of strong attraction are arrested at the interfacial width. Taken together, we thus demonstrated that interactions can affect patterns substantially. Our linear stability analysis presented in the electronic supplementary material and a recent pre-print [36] demonstrate that these results do not depend on the specific choice of the reactions. Instead, interactions can generally lift restrictions on diffusivities and reaction nonlinearities imposed by ordinary Turing patterns. Since physical interactions are virtually always present, many natural patterns can probably be explained by similar mechanisms.

Physical interactions in natural systems can stem from various sources and are virtually unavoidable in multi-component systems. We need to investigate such systems in more detail, both in terms of physical interactions [30], chemical reaction networks [11,37], and conservation laws [38]. For instance, Turing patterns can form when two species have equal diffusivity, while a third one is immobile [39], to produce effective differences in diffusivities. Explaining natural patterns in detail also requires incorporating growth [40], flows [41], noise and delays [10]. Moreover, natural patterns often form in complex geometries, including coupled layers [42] and curved surfaces [43,44], where the mechano-chemical coupling [45] can lead to dynamic patterns [46]. The organization of biological cells is a particularly exciting example since biomolecules are known to interact and react [14]. While this sometimes leads to spatial patterns explained by Turing’s mechanism [7], other examples are akin to active droplets [21]. Another possibility is patterns formed by self-propelled agents, which can exhibit motility-induced phase separation [47] and explain some population patterns successfully [16]. In all these cases, physical interactions will affect patterns qualitatively and quantitatively, opening new perspectives on how natural patterns emerge.

## 4. Methods

We perform numerical simulations of equations (2.3) and (2.4) on an equidistantly discretized grid using second-order finite-differences to approximate differential operators [48]. We evaluate $\nabla \mu_{i}$ on a staggered grid to ensure material conservation and use an explicit Euler scheme for the time evolution.

## Data Availability

Code needed to reproduce the results is available from the Zenodo repository: [49] https://doi.org/10.5281/zenodo.8068835 and from the GitHub repository: https://github.com/zwicker-group/paper-non-ideal-turing-patterns. All other data are available in the manuscript or the electronic supplementary material [50].
